# Intérêt de la cytoponction à l'aiguille fine dans le diagnostic des tumeurs parotidiennes

**DOI:** 10.11604/pamj.2019.33.65.18259

**Published:** 2019-05-29

**Authors:** Rachida Bouatay, Rim Ben Nasr, Adnène Moussa, Amel El Korbi, Khaled Harrathi, Jamel Koubaa

**Affiliations:** 1 Service d'Oto-rhino-laryngologie et de Chirurgie Cervico-faciale de l'Hôpital Universitaire Fattouma Bourguiba de Monastir, Tunisie; 2 Service d'Anatomie Pathologique de l'Hôpital Universitaire Fattouma Bourguiba de Monastir, Tunisie

**Keywords:** Cytoponction, tumeurs parotidiennes, sensibilité, spécificité, Fine needle aspiration biopsy, parotid tumors, sensitivity, specificity

## Abstract

L'objectif de ce travail était de déterminer la valeur diagnostique de la cytoponction à l'aiguille fine (CPAF) et son apport dans la prise en charge des tumeurs parotidiennes, à travers une étude rétrospective portant sur 47 patients opérés d'une tumeur parotidienne et ayant eu une cytoponction préopératoire. Quatre-vingt-un pourcent des malades présentaient une tumeur bénigne et 19% une tumeur maligne. La sensibilité et la spécificité de la CPAF étaient respectivement de 78% et de 92%. Les tumeurs parotidiennes ont été correctement classées comme malignes ou bénignes dans 89% des cas, le taux d'exactitude globale était de 64,4%. La CPAF est un examen fiable permettant une information préopératoire sur le planning thérapeutique et les suites postopératoires.

## Abstract (en)

This study aimed to determine the diagnostic value of fine needle aspiration biopsy (FNAB) and of its contribution to the management of parotid tumors. We conducted a retrospective study of 47 patients who had undergone parotidectomy as well as preoperative fine needle aspiration biopsy. The study highlighted that eighty one percent of patients had a benign tumor while 19% of patients had a malignant tumor. The sensitivity and specificity of FNAB were 78% and 92% respectively. Parotid tumors were correctly classified as malignant or benign in 89% of cases, overall accuracy was 64.4%. FNAB is a reliable examination providing preoperative informations about the treatment plan and the postoperative course.

## Introduction

Les tumeurs des glandes salivaires représentent 3% à 5% des tumeurs de la tête et du cou. Elles ont une localisation essentiellement parotidienne (90%) et sont caractérisées par une grande diversité histologique, pouvant parfois poser des difficultés diagnostiques [1]. La cytoponction ne fait pas l'unanimité dans la prise en charge de la pathologie parotidienne où ses indications et l'interprétation de ses résultats suscitent encore des controverses. Le but de ce travail est d'évaluer, à travers notre série avec revue de la littérature, la valeur diagnostique de la cytoponction afin de déterminer sa place, ses avantages et ses limites dans la prise en charge d'une tumeur parotidienne.

## Méthodes

Il s'agit d'une étude rétrospective portant sur 47 patients opérés d'une tumeur parotidienne sur une période de 10 ans (2006 - 2015). Il a été inclus dans cette étude tous les patients ayant eu une cytoponction préopératoire.

## Résultats

**Figure 1:**
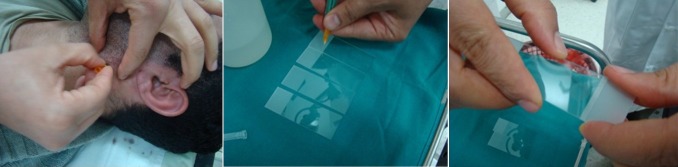
Technique de la cytoponction

Durant la période d'étude, 121 patients ont eu une parotidectomie pour une tumeur parotidienne. On a inclus 47 cas opérés d'une tuméfaction parotidienne et ayant eu une cytoponction antérieure (39%). La technique de la cytoponction est illustrée dans la Figure 1. Il s'agissait de 38 tumeurs bénignes (81%) et de 9 tumeurs malignes (19%). Tous nos malades ont présenté une symptomatologie commune représentée par une tuméfaction parotidienne. La cytoponction s'est avérée non contributive chez 2 patients soit dans 4% des cas. Aucune complication secondaire à la cytoponction n'a été notée. Les résultats de la cytoponction sont résumés dans le Tableau 1. L'étude microscopique définitive des pièces opératoires a révélé une grande diversité histologique largement dominée par l'adénome pléomorphe (40%), rejoignant ainsi les résultats cytologiques (Tableau 2). L'examen extemporané a été réalisé dans tous les cas. Ses données étaient concordantes avec celles des pièces d'exérèse sauf pour 2 malades où la confusion a été faite entre un papillome intra-canalaire et un adénocarcinome et, entre une lésion bénigne et un carcinome épidermoïde. Les résultats de l'examen cytologique comparés aux résultats définitifs histologiques sont résumés dans le Tableau 3.

**Tableau 1:** Distribution des lésions bénignes et malignes

Diagnostic cytologique	N	pourcentage %
**Bénin**	35	75
Adénome pléomorphe	21	44,7
Tumeur de Warthin	6	12,7
Kyste branchial	1	2,1
Lymphadénite tuberculeuse	1	2,1
Hyperplasie lymphoïde réactionnelle	2	4,3
Mycobactériose atypique	1	2,1
Lésion kystique bénigne	1	2,1
Bénin indéterminé	2	4,3
**Malin**	10	21
Carcinome ductal Carcinome à cellules acineuses	1	2,1
Carcinome mucoépidermoide	1	2,1
Carcinome peu différencié	2	4,3
Métastase d’un mélanome	1	2,1
Métastase d’un carcinome papillaire de la	1	2,1
thyroïde	2	4,3
Malin indéterminé	2	4,3

**Tableau 2:** Répartition histologique des lésions parotidiennes bénignes

Diagnostic histologique	Effectifs	Pourcentage %
***Lésions bénignes***		
Adénome pléomorphe	**19**	**40,4**
Tumeur de Warthin	**14**	**29,8**
Kyste salivaire	**2**	**4,3**
Kyste lymphoépithélial	**1**	**2,1**
Hyperplasie lymphoïde réactionnelle	**1**	**2,1**
Lymphadénite tuberculeuse	**1**	**2,1**
***Lésions malignes***		
Adénocarcinome sans autre indication	**1**	**11,1**
Carcinome à cellules acineuses	**1**	**11,1**
Carcinome adénoïde kystique	**1**	**11,1**
Carcinome mucoépidermoïde	**2**	**22,2**
Lymphome	**1**	**11,1**
Carcinome épidermoïde	**2**	**22,2**
Métastase d'un mélanome	**1**	**11,1**

**Tableau 3:** Corrélation: cytologie / histologie définitive

Histologie/Cytologie	Bénin	Malin	Total
Bénin	33	2	35
Malin	3	7	10
Indéterminée	2	0	2
Total	37	9	47

La cytoponction était non contributive dans deux cas (un cas d'adénome pléomorphe et un cas de tumeur de Warthin) par insuffisance du matériel cellulaire. Parmi les 45 cytoponctions analysées, le diagnostic était exact dans 89% des cas (40 cas). Le résultat était faussement négatif dans 2 cas (6%) et faussement positif dans 3 cas (30%). La sensibilité calculée pour le diagnostic de malignité était de 78% et la spécificité de 92%. La valeur prédictive positive était de 70% et la valeur prédictive négative de 94,3% (Tableau 4). L'adénocarcinome sans autre indication et le carcinome adénoïde kystique étaient les deux types histologiques responsables de faux négatifs (diagnostiqués en tant qu'adénomes pléomorphes à la cytologie). La tumeur de Warthin était le type histologique responsable de faux positifs dans trois cas. Dans notre série, sur 45 cytoponctions, un typage histologique de la tumeur a pu être rendu dans 64,4% des cas. Pour les tumeurs bénignes, l'exactitude du typage histologique de la tumeur était de 72%. Pour les tumeurs malignes, le typage histologique était exact dans 33% des cas. Dans notre série, l'examen cytologique était concordant avec l'examen extemporané dans 84%, la sensibilité et la spécificité étaient respectivement de 71% et de 87%. Le taux de faux négatifs était estimé à 5,7% (2cas) et le taux des faux positifs à 50% (5 cas). La valeur prédictive positive était de 50% et la valeur prédictive négative de 94,2%.

**Tableau 4:** Performances de l’examen cytologique pour le diagnostic de malignité

		[Intervalle de Confiance 95%]
**Sensibilité**	78%	[40,19%-96,05%]
**Spécificité**	92%	[76,40%-97,82%]
**VPP**	70%	[39,67%-89,22%]
**VPN**	94,28%	[81,39%-98,41%]
**Efficacité**	89%	
**RVP**	9,33	[2,99-29,13]
**RVN**	0,24	[0,07-0,82]

VPP: valeur prédictive positive, VPN: valeur prédictive négative, RVP: rapport de vraisemblance positive, RVN: rapport de vraisemblance négative

## Discussion

La CPAF prend une place clé dans l'exploration initiale des tumeurs des glandes salivaires en particulier de la glande parotide compte tenu de leur grande diversité histologique. C'est un geste aisé, dont la technique est peu coûteuse et le résultat peut être obtenu en quelques minutes. C'est un acte qui n'est pas invasif et ses complications sont mineures, l'anesthésie n'est pas nécessaire car la douleur occasionnée est équivalente à celle de la ponction elle-même. L'interprétation des prélèvements cytologiques des tuméfactions parotidiennes s'oppose à des difficultés de caractérisation, liées en partie à la grande diversité lésionnelle, et d'autre part à leur composition complexe. De ce fait, il est recommandé de réaliser une approche diagnostique par étapes afin d'établir un cytodiagnostic approprié de ces lésions [2, 3]. Cette approche consiste à distinguer les lésions d'origine salivaire des celles des tissus adjacents, déterminer s'il s'agit d'une lésion néoplasique ou non, distinguer les tumeurs bénignes des tumeurs malignes, et de préciser le grade si possible (bas ou haut grade) pour ces dernières. Le type histologique peut être proposé en cas de certitude diagnostique mais sera parfois mieux laissé à l'histologie définitive.

L'utilité de la cytoponction dans la prise en charge des tumeurs parotidiennes reste controversée. En réalité, la majorité des chirurgiens ne planifient pas leur acte chirurgical en fonction des résultats de la cytoponction, mais proposent une parotidectomie avec examen extemporané et modifient leur attitude (totalisation, curage ganglionnaire complémentaire) en fonction des résultats peropératoires. Les études récentes, ont démontré que la connaissance préopératoire de la nature bénigne ou maligne d'une tuméfaction parotidienne aurait un impact évident pour planifier convenablement la prise en charge ultérieure (Traitement médical, surveillance, bilan préopératoire, rendez-vous et type de chirurgie) [3]. Il est de plus en plus évident que la cytoponction couplée à l'imagerie, notamment l'imagerie par résonance magnétique (IRM) avec l'utilisation des nouvelles séquences (séquences de perfusion et de diffusion), occupe une place prépondérante dans la prise en charge des nodules parotidiens [4]. Elle permet ainsi d'effectuer la sélection des patients pour lesquels la chirurgie est indiquée. Elle permet au chirurgien de définir la stratégie chirurgicale et d'informer le patient des modalités du traitement [5].

La connaissance préopératoire de la nature maligne de la tumeur changerait les suites opératoires [6]: elle augmente de manière significative le nombre de curages ganglionnaires concomitants ainsi que le nombre de marges saines à l'examen histologique de la pièce d'exérèse, elle diminue les chirurgies de rattrapage et elle améliore le succès de la prise en charge chirurgicale initiale et influence la survie à long terme [3]. A contrario, O'Brien estimait que la cytoponction à l'aiguille fine ne devrait être recommandée que lorsque les résultats de l'examen cytologique pourraient changer la prise en charge [7]: valider une approche non chirurgicale (état général du malade précaire, population pédiatrique, métastases et lymphomes), doute sur la nature intra-parotidienne de la tuméfaction, evaluation d'une masse para-pharyngée dont le diagnostic de malignité peut changer la voie d'abord chirurgicale et lorsque la tuméfaction paraît cliniquement maligne et qu'un sacrifice nerveux semble être nécessaire.

La méta-analyse réalisée par Schmidt *et al.* en 2011 et regroupant 64 études et plus de 6000 patients confirme les excellentes performances de la cytoponction qui restent tributaires de l'expérience du préleveur et du cytopathologiste. Quand ces exigences sont remplies, la cytoponction peut être considérée comme un examen intéressant et fiable [8]. Le taux des cytoponctions non contributives présente un des principaux inconvénients de la technique. Il varie de 3 à 34% en fonction des séries [4, 5]. Ce taux est estimé à 8,6% dans la méta-analyse de Schmidt *et al.* [8]. Dans notre étude seulement 4% des cytoponctions étaient non contributives. Cela pourrait être expliqué par l'hétérogénéité très fréquente des tumeurs salivaires et la difficulté d'obtenir un matériel cellulaire significatif: variabilité tissulaire, présence de zones kystiques ou nécrotiques (tumeur de Warthin, carcinome muco-épidermoïde), prélèvements hémorragiques ou paucicellulaires. Le guidage échographique prend alors toute sa place, y compris dans les lésions aisément palpables, permettant de prélever sélectivement les zones tissulaires, plus informatives sur le plan cytologique [3, 9]. La réalisation de la cytoponction par un préleveur entrainé améliore aussi ces performances et diminue le taux de cytoponctions non contributives [4, 9].

Dans notre série toutes les cytoponctions ont été réalisées sans guidage échographique. Le taux de faux négatifs dans notre étude était de 6% (2 cas): il s'agissait d'un cas d'adénocarcinome sans autre indication et d'un cas de carcinome adénoïde kystique qui étaient faussement diagnostiqués comme adénomes pléomorphes. Ce taux atteint 20% dans la méta-analyse de Schmidt *et al.* [8]. Ces erreurs diagnostiques sont fréquentes dans le cas des tumeurs malignes de bas grade (les critères de malignité classiques font défaut, et le diagnostic repose sur l'infiltration qui n'est pas visible en cytologie) et dans les lésions kystiques pauci cellulaires rendant leur interprétation difficile [9, 10]. L'étude des séries rapportées a montré que les types histologiques les plus fréquemment responsables de faux négatifs sont le carcinome mucoépidermoïde de bas grade, le carcinome à cellules basales et les lymphomes à petites cellules [3, 11, 12]. Trois tumeurs de Warthin étaient faussement classées comme tumeurs malignes par la cytoponction (faux positifs), ce résultat est concordant avec les données de la littérature et serait dû à la fréquence des atypies régénératives et de la métaplasie malpighienne dans ce type de tumeur [12]. Les adénomes pléomorphes cellulaires sont également à l'origine de nombreux faux diagnostics positifs. Le typage histologique des adénomes pléomorphes était exact dans 18 sur 19 cas (95%), ce résultat s'accorde avec les données rapportées dans la littérature qui varient de 82 à 94% [3].

Plusieurs auteurs s'accordent que le diagnostic de lymphome est difficile par la cytoponction et qu'une confirmation histologique s'avère souvent indispensable [9, 13]. Il s'agit dans la plupart des cas d'un lymphome MALT (Mucosa-Associated Lymphoid Tissue) développé sur une sialadénite lymphoépithéliale. Si le diagnostic de lymphome est suspecté, la cytométrie de flux et la biologie moléculaire peuvent être utiles. Dans notre série le seul cas de lymphome était à grandes cellules et a été diagnostiqué en cytologie comme un carcinome peu différencié. L´étude des principales séries publiées [4, 8, 14-18] (Tableau 5) fait apparaître que la sensibilité de la cytoponction varie de 54 à 92%, et que sa spécificité varie de 86 à 100% [4, 8]. Une méta-analyse réalisée en 2011 retrouvait une sensibilité globale estimée à 80% et une spécificité estimée à 97% [8]. Certains auteurs ont expliqué cette sensibilité faible par un taux élevé de faux négatifs pour le diagnostic de malignité, la tumeur étant faussement classée bénigne dans 8 à 46% de cas en fonction des séries.

**Tableau 5:** Récapitulatif des études sur la cytoponction parotidienne dans la différenciation bénin/malin

	N	Se (%)	Sp (%)	Efficacité (%)	VPP (%)
Stewart *et al.* [14]	341	**92**	100	98	
Zbaren *et al.* [15]	228	64	95	86	83
Bajaj *et al.* [16]	69	84	96	94	
Aversa *et al*. [17]	310	83	**100**	97	
Schmidt *et al*. (méta-analyse) [8]	6169	80	97		90
Fakhry *et al.* [4]	202	80	89,5	86,5	73
Gudmundsson *et al.* [18]	114	73	97		
**Notre série**	**47**	**78**	**92**	**89**	**70**

Dans notre étude, la sensibilité et la spécificité étaient respectivement de 78% et 92%. Les tumeurs parotidiennes ont été correctement classées comme malignes ou bénignes dans 89% des cas, ce qui est en accord avec les données de la littérature. Les résultats pour le typage histologique des tumeurs par la cytoponction varient de 29 à 84% en fonction des séries [11]. Dans notre série, parmi les 45 cas où le typage a été possible, le taux d'exactitude globale était de 64,4% des cas. Ce taux était de 72% pour les tumeurs bénignes et de 33% pour les tumeurs malignes. Ce taux faible pour les tumeurs malignes retrouvé dans la majorité des séries de la littérature serait dû à la rareté des tumeurs malignes et à leur diversité histologique. Le typage histologique des tumeurs primitives de la parotide est un exercice difficile et nos résultats sont comparables à ceux publiés dans la littérature. Cela peut s'expliquer par le fait que les tumeurs des glandes salivaires sont relativement rares et très polymorphes. Si ce polymorphisme oriente vers le diagnostic en histologie, il s'avère souvent trompeur en cytologie [19]. Ainsi, il existe une confusion dans l'interprétation des résultats, une impossibilité de les comparer d'une institution à une autre avec impossibilité d'assigner à chaque diagnostic un risque de malignité et une conduite à tenir appropriés. Ceci a conduit un groupe d'experts (cytopathologistes et histopathologistes) à élaborer un système unifié de réponse cytologique avec une terminologie standardisée et une stratification du risque de malignité à l'instar des autres classifications déjà adoptées (col utérin, thyroïde, tractus urinaire et pancréas).

Cet effort de standardisation a commencé en septembre 20^15^ au Congrès Européen de Cytologie qui a été tenu à Milan, Italie, sous la tutelle de l'*American Society of Cytopathology* et l' *International Academy of Cytology*. Le groupe d'experts a proposé un schéma de classification comportant 6 catégories et devra l'entériner avant janvier 2017 [20]: non-diagnostique, non-néoplasique, atypie de signification indéterminée, néoplasique, Bénin, potentiel de malignité incertain, suspect de malignité et malin. Une fois validée par des preuves basées sur l'évidence, ce système permettra d'améliorer la communication entre pathologiste et clinicien, de faciliter la corrélation cyto-histologique ainsi que le partage des données entre différentes institutions et les études multicentriques [20].

## Conclusion

Nos résultats nous ont amené à conclure que la cytoponction est un examen peu invasif, peu couteux et fiable avec une bonne sensibilité permettant une meilleure planification du geste chirurgical dans son délai, et une information préopératoire plus adéquate influant sur le planning thérapeutique et les suites postopératoires. De ce faite, elle s'intègre dans le bilan initial des tumeurs des glandes salivaire. Cependant, elle ne saurait guider à elle seule l'attitude chirurgicale puisqu'elle constitue avec la clinique et l'imagerie notamment l'IRM le trépied fondamental dans la prise en charge de tumeurs parotidiennes.

### État des connaissances actuelles sur le sujet

- La grande diversité histologique des tumeurs parotidiennes posant des problèmes diagnostiques et thérapeutiques;
- La place de la cytoponction dans la prise en charge de ces tumeurs reste jusqu'à ce jour controversée;
- Et même l'interprétation des résultats de la cytoponction suscite encore des controverses et n'est pas uniforme.

### Contribution de notre étude à la connaissance

- La cytoponction est un examen fiable pour le diagnostic de malignité des tumeurs parotidiennes avec une sensibilité de 78% et une spécificité de 92%;
- Dans notre série, la cytoponction s'intègre dans le bilan initial des tumeurs parotidiennes et constitue avec la clinique et l'IRM le trépied fondamental;
- Une standardisation des résultats serait utile pour la stratification du risque de malignité.

## Conflits des intérêts

Les auteurs ne déclarent aucun conflit d'intérêts.
